# Dual inhibition of FGFR4 and BCL-xL inhibits multi-resistant ovarian cancer with BCL2L1 gain

**DOI:** 10.18632/aging.203386

**Published:** 2021-08-05

**Authors:** Ting Guo, Chao Gu, Bin Li, Congjian Xu

**Affiliations:** 1 Department of Gynecology, Obstetrics and Gynecology Hospital of Fudan University, Shanghai, P.R. China

**Keywords:** ovarian cancer, BCL2L1, resistance, copy number

## Abstract

Aim: Overexpression of BCL2L1 (BCL-xL) was associated with platinum resistance in ovarian cancer (OvCa). However, role of copy number (CN) gain of BCL2L1 in OvCa remains elusive.

Methods: *In silico* analyses of multiple public datasets were perform. Validation was carried out in our tissue microarray (TMA) of OvCa cases. *In vitro* and *in vivo* assays was performed to explore potential targeted compound against BCL2L1-gained OvCa.

Results: BCL2L1 was gained in ~60% of OvCa. BCL2L1 was differentially expressed between healthy and cancerous ovarian cases. BCL2L1 gain was not prognostic either in overall or in progression-free survival but higher BCL2L1 expression was associated with worsened survival, indicating biological distinction between CN gain and overexpression of the gene. BCL2L1 gain was associated with multi-resistance to various drug with no significant sensitivity to any single agent. Only CRISPR-mediated BCL2L1 knockout, but not shRNA could be inhibitive. Combined genetic silencing of FGFR4/NCAM and BCL2L1 with shRNA induced potent inhibition of BCL2L1-gained OvCa with durable effect. Combined inhibition of FGFR/BCL-xL was required for inhibiting BCL2L1-gained OvCa *in vitro* and *in vivo*. Only dual inhibition of FGFR/BCL-xL without platinum was tolerable *in vivo*.

Conclusion: Gain of BCL2L1 is associated with resistance to multiple anti-cancer agents in OvCa. Dual inhibition of FGFR4 and BCL-xL showed potent effect and tolerable toxicity, holding promise to further translation.

## INTRODUCTION

Ovarian cancer (OvCa) is a major cause of death in women. In 2019, 22,500 newly diagnosed cases and approximately 14,000 deaths occurred in the United States alone [1]. Although curative surgery and platinum-based chemotherapy play critical roles in treatment, more than 80% of cases face relapse. Therefore, unveiling other the underlying genetic dispositions to OvCa development is critical therapeutically.

Copy number variation (CNV) plays a role in OvCa. The release of the sequence data of The Cancer Genome Atlas (TCGA) for high-grade serous ovarian cancer (HGSOC) has enabled the genetic and genomic landscape of OvCa to be analyzed more comprehensively strategy [2]. The currently established CNV includes the gain of Cyclin E1 (CCNE1). However, genes within other amplicons that may also play critical roles in OvCa are less frequently reported, particularly the 20q11.21 region encompassing BCL2L1. BCL2L1 is one of the most common amplified genes among cancers. It encodes the antiapoptotic protein BCL-xL, which belongs to the antiapoptotic BCL2 family. Increased genetic dosage of one or several members of the BCL2 family has been associated with a worsened outcome in various cancers [3–5]. BCL2L1 overexpression is associated with resistance to platinum and PARP inhibitors. However, CN gain/amplification may not exert the same biological function as overexpression.

Our group conducted a series of studies focusing on CNV in OvCa [6–9], particularly the role of focal gain of 20q11.21. In the current study, we used OvCa cells with different CNs of BCL2L1 and examined the drug sensitivity profile *in vitro* and *in vivo*. We found that BCL2L1 gain is common in OvCa and exhibits a distinct biology compared with overexpression, showing high conservative BCL2L1 expression. Only dual inhibition of FGFR4 and BCL-xL showed potent effects and tolerable toxicity, holding promise for further translation.

## MATERIALS AND METHODS

### *In silico* analysis

Reproduction of the CPTAC ovarian cancer dataset was performed using the UALCAN online platform (http://ualcan.path.uab.edu/index.html). Reproduction of the TCGA OvCa dataset was performed using the cBioPortal online platform (http://www.cbioportal.org/) with the selection of Firehose Legacy subsets [10, 11]. A copy number variation (CNV) dataset was used with a GISTIC value of >2 designated as amplification and between 1 and 2 designated as gain. BCL2L1 was queried to profile focal CNVs of 1q25.3 across all TCGA cancer types. Gene enrichment analysis for BCL2L1-gained cases was analyzed using the GSEA approach with mRNA expression data (RNA seq V2) retrieved from TCGA [12]. Correlations of mRNA expression in tumor tissues were also analyzed and plotted using cBioPortal. Correlations of mRNA expression in OvCa cell lines were analyzed and plotted using the DepMap Portal online platform (https://depmap.org/portal/), which integrated genomic data from various high-throughput sequence resources. Log-rank survival analysis for BCL2L1 was performed using cBioPortal for CNV and TIMER (https://cistrome.shinyapps.io/timer/) for expression. Cox regression was performed using TIMER. The Harmonizome platform was used to examine the copy number of BCL2L1 in OV cell lines (http://amp.pharm.mssm.edu/Harmonizome/).

### Tissue microarray (TMA) and immunohistochemistry (IHC)

A commercially available deidentified TMA chip of 80 OvCa sections and 10 normal controls were used [13]. The sections were deparaffinized after incubation for 30 min. Before graded dehydration, the sections were immersed in xylene for 15 min and 1:1 xylene and alcohol for 10 min. Hydrogen peroxide (3%) was used for blockade at room temperature. Antigen recovery was prepared in 0.01 M sodium citrate buffer solution (pH 6.0) in a microwave for 20 min. After cooling, 10% serum in TBS was used for blockade. The primary antibody against BCL2L1 (AHO0222; 1:200; Thermo Fisher) was applied overnight. The corresponding secondary antibody was applied, and IHC scoring was calculated as previously reported [13].

### Cell culture and RNA interference (RNAi)

Based on Harmonizome, SK-OV-3 was selected as the BCL2L1-amplified OvCa cell line, OVCAR433 was selected as the BCL2L1-overexpressing cell line, and A2780 was selected as the BCL2L1-underexpressing cell line. All the cells were archived lines in our laboratory. The cells were cultured in RPMI-1640 medium supplemented with 10% FBS. The GPP Web Portal (https://portals.broadinstitute.org/gpp/public/) was used for shRNA construction targeting BCL2L1, NCAM and FGFR4. The cDNA clone for BCL2L1 was obtained from Origene. Overexpression was realized by lentiviral delivery using a polybrene system. Quantitative PCR was performed to examine the shRNA effect and constitutive BCL2L1 expression level. The primers were constructed using PrimerBank (https://pga.mgh.harvard.edu/primerbank/). The treatment details of A-1331852 (A; BCL-xL inhibitor), BLU9931 (B; FGFR4 inhibitor) and cisplatin (Pt; 1 mg/mL in 0.9% NaCl) are indicated in the Figure legends of different assays.

### Western blotting

A standard protocol of western blotting was performed. Briefly, cells were lysed using RIPA buffer, and total protein was extracted. After the concentration was determined, the protein was loaded with buffer onto SDS-PAGE gels with subsequent electrophoresis. The protein was then transferred to a PVDF membrane, which was blocked with 5% nonfat milk. Primary antibodies against BCL2L2 (BCL-w) (ab190952; 1:200; Abcam), BCL2L1 (BCL-xL) (ab270253; 1:200; Abcam), FGFRs (ab76464 for FGFR1 at 1:500; ab109372 for FGFR2 at 1:500; ab133644 for FGFR3 at 1:500; ab178396 for FGFR4 at 1:500; Abcam) and NCAM1 (ab9272; 1:500; Abcam) were applied overnight. The corresponding secondary antibodies and ECL were routinely applied. Densitometry was analyzed using ImageStudio software.

### Proliferation viability assay

Cell proliferation was profiled using an EdU staining kit according to the manufacturer’s protocol. Cell viability was studied using the MTT assay. Briefly, cells were seeded in 96-well plates and analyzed in a plate reader for absorbance at each time point of interest.

### Flow cytometry

The FASCanto flow cytometry system was used to measure the cell cycle and apoptotic profiles. For cell cycle analysis, cells were fixed using cold ethanol and later treated with cell cycle staining buffer. For apoptosis, Annexin V was applied to cells, and apoptotic cells were defined as the sum of early and late apoptotic cells.

### Colony formation

Seventy-two hours after viral infection, approximately 400-1000 cells were seeded in each well of a 6-well plate. Medium was changed every 3 days. The cells were fixed with 4% methanol on day 11 and subsequently stained with crystal violet.

### CRISPR/Cas9 assay

A standard CRISPR assay was applied [14]. sgRNA sequencing for BCL2L1 was designed using the CRISPRko platform (https://portals.broadinstitute.org). Three pairs of sequences with the highest pick scores were tested, among which there were 2 sense and 3 antisense sequences.

### Wound healing assay

Cells were seeded at a density of 7 × 10^3^ cells on each side of a culture insert using a 500-μm separation between each side of the well and allowed to grow for 24 h in growth medium. After removal of the insert, the cells were incubated in the same medium. The cells were photographed using phase contrast microscopy at insert removal (0 h) and following 6 and 24 h of incubation.

### Transwell assays

Both invasion and migration were measured using the Transwell assay. Cells were seeded in the upper chamber of the Transwell plate at a density of 1×10^6^/ml, either coated (for invasion) or uncoated (for migration) with Matrigel. The upper chamber was supplemented with serum-free media, while the lower chamber was filled with complete medium. Cells that penetrated were stained with crystal violet and counted.

### Xenograft mouse model

A subcutaneous tumor implantation mouse model was established. Approximately 10^7^ SK-OV-3 cells were injected s.c. in the axillary region of 10 female mice at 4 weeks of age per group. Tumors were calibrated every 4 days (checkpoint), and mice were euthanized on the fifth checkpoint unless tumors reached 2000 mm^3^ in size calibrated using the formula Length * Width^2^ * 0.523. The mice were treated during the light cycle with 25 mg/kg/d A-1331852 and 100 mg/kg of BLU9931 twice daily for 14 days by daily oral gavage. The cisplatin dosage represented the mouse dose equivalent of 75 mg/m^2^ of dose in humans and was administered as 3 treatments, every 2 weeks, starting 14 days after tumor implantation. All the procedures were approved by the Fudan Animal Welfare and Ethics Reviewer Board.

### Statistical analysis

Statistical analyses were performed using GraphPad Prism 7.0 for Mac. Comparisons between two groups were studied using the Mann-Whitney test for nonparametric variants (IHC scores) and Student’s t test for parametric variants (RNA-seq reads). The survival data were presented using the Kaplan-Meier curve and were compared using the log-rank test. A P value < .05 was accepted as significant.

## RESULTS

### BCL2L1 is frequently gained in OvCa

**Figure 1:**
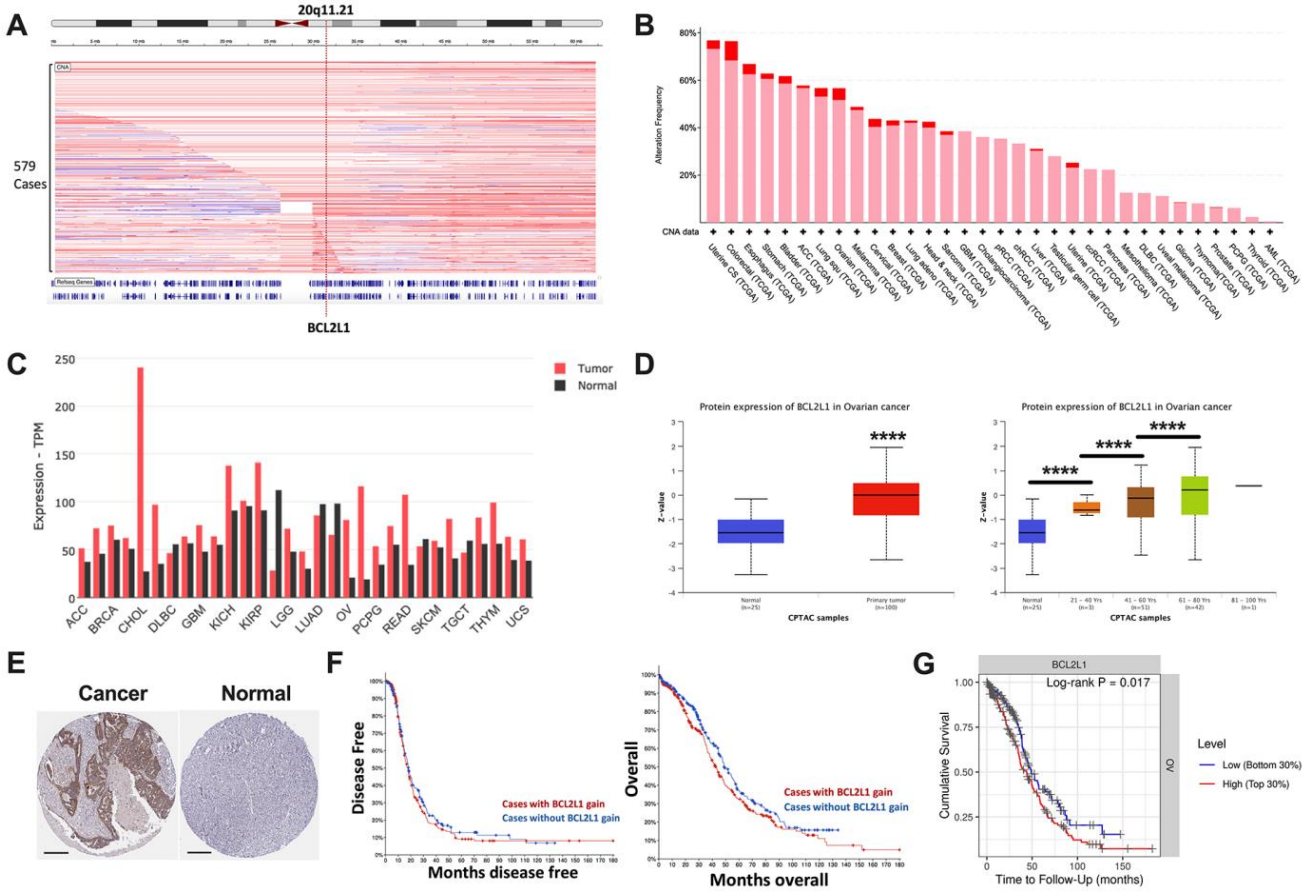
**BCL2L1 is frequently gained in OvCa.** Reproduced from TCGA OvCa dataset, shown were (**A**) copy number alteration of Cytoband 20q11.21 that encompassed BCL2L1; (**B**) frequency of gain (pink) and amplification (red) of BCL2L1 across all cancers in TCGA dataset; (**C**) differential expression of BCL2L1 across TCGA cancers; (**D**) Reproduced from CPTAC dataset, shown were protein level of BCL2L1 in different clinicopathological parameters; (**E**) representative IHC images of BCL2L1 in normal ovary tissue and ovarian cancer tissue; (**F**) overall and progression-free survival of OvCa cases with or without BCL2L1 gain/amplification in TCGA cohort; (**G**) overall survival of OvCa cases with top 30% or bottom 30% of BCL2L1 expression in TCGA cohort (*P < .05; **P < .01; ***P < .001; ****P < .0001).

Focal gain at 20q11 occurred frequently in OvCa cases in the TCGA cohort (Figure 1A). BCL2L1 located on 20q11.21 was recurrently gained in ~60% of ovarian cancer cases, ranking among the top 10 of all TCGA cases (Figure 1B). BCL2L1 was differentially expressed between healthy and cancerous ovarian cases, showing substantially higher expression in ovarian cancer (Figure 1C). BCL2L1 protein was also differentially expressed in OvCa, as demonstrated by both mass spectrometry and IHC (Figure 1D, 1E). Interestingly, although BCL2L1 expression was not associated with tumor grade or stage (Table 1), it was significantly higher in older patients (Figure 1D). However, in the multivariate model, only age, but not BCL2L1 expression, remained significant (Table 2). As expected, BCL2L1 gain was not prognostic either in overall or progression-free survival (Figure 1F), but higher BCL2L1 expression was associated with a worsened survival, indicating a biological distinction between CN gain and overexpression of the gene (Figure 1G).

**Table 1:** Associations between clinicopathological parameters and BCL2L1 expression in ovarian cancer tissue microarray (SE =).

	***N***	**BCL2L1**		
		**Median**		**SE**
**Tissue**				
Normal	10	0	±	0.1528
Cancer	80	1	±	0.1375
*P value*		< 0.001		
**Stage**				
T1	49	1	±	0.2123
T2	17	2	±	0.2647
T3	14	1	±	0.2891
*P value*		0.8775		
**Lymph node**				
N0	67	1	±	0.1648
N1	13	1	±	0.3608
*P value*		0.8138		
**Lesion**				
Primary	80	1	±	0.1492
Metastatic	10	1	±	0.3416
*P value*		0.9366		
	Median ± SE	***Spearman R***		***Linear P***
**Age (year)**	59 ± 1.07	0.012		0.9077

**Table 2:** Multivariate analysis of prognostic role of BCL2L1 reproduced from the TCGA-HGSOC dataset.

	**Coefficient**	**HR**	**95% CI**		**P value**
			**Lower**	**Upper**	
**Race_Black**	0.358	1.431	0.558	3.671	0.456
**Race_White**	-0.04	0.961	0.425	2.172	0.924
**Age**	0.024	1.024	1.013	1.035	<0.001
**Tumor purity**	-0.29	0.748	0.303	1.847	0.53
**BCL2L1**	0.208	1.231	0.946	1.603	0.122

### BCL2L1 gain is associated with multiresistance

**Figure 2:**
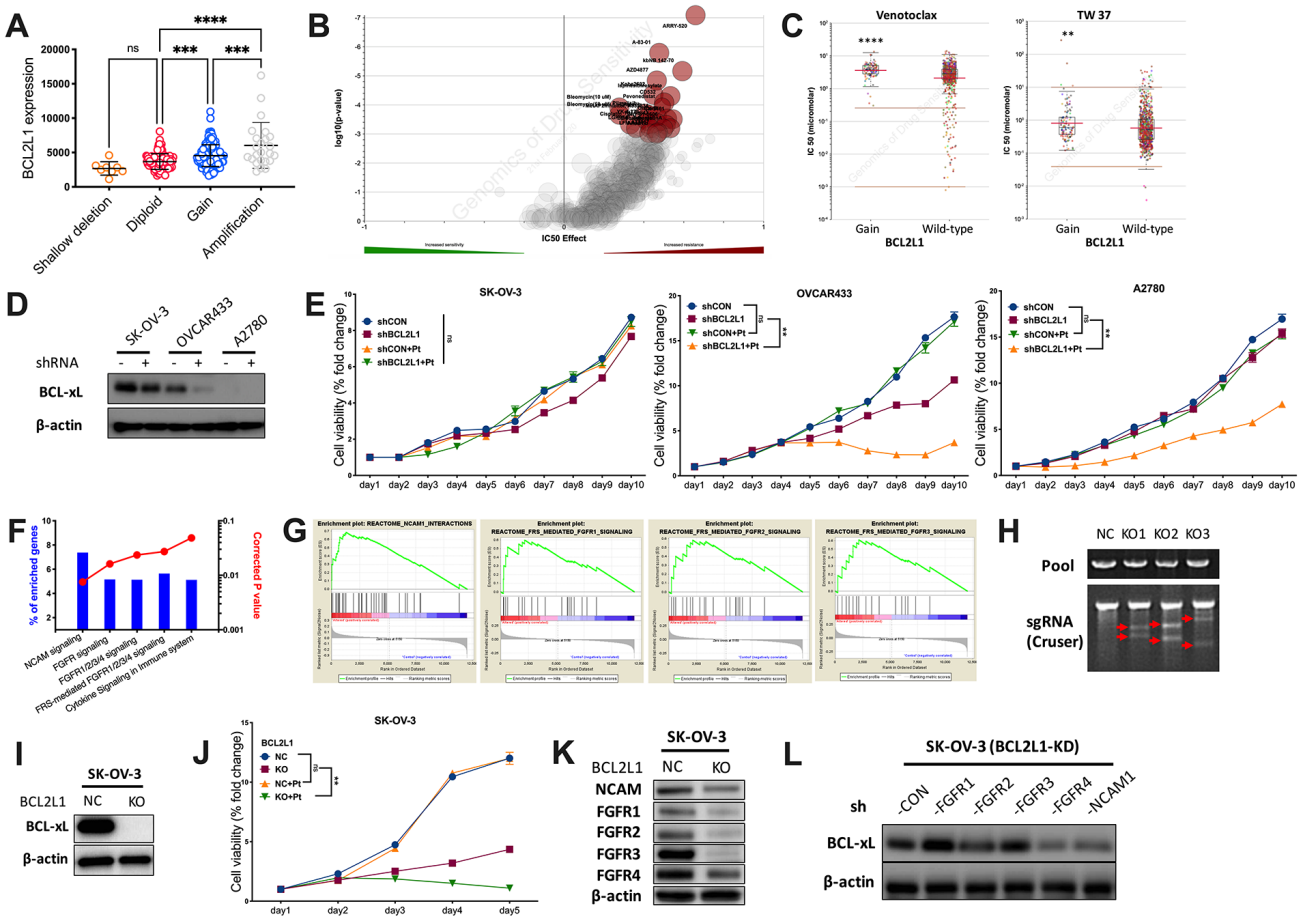
**BCL2L1 gain is associated with multi-resistance.** Reproduced from TCGA OvCa dataset, shown were (**A**) BCL2L1 expression in different copy number alteration in OvCa; Reproduced from GDSC dataset, shown were (**B**) volcano plot revealing drug sensitivity of cancer cells with 20q11.21 gain; (**C**) cancer cells with BCL2L1 gain showing resistance to 2 BCL2-targeted compounds; (**D**) western blotting showing BCL-xL level in 3 OvCa cell lines with different BCL2L1 status treated or untreated with BCL2L1 knockdown (KD) by shRNA; (**E**) cell viability detected by MTT assay in 3 OvCa cell lines over 10-day course with or without BCL2L1-KD plus cisplatin (Pt, 1 mg/mL in 0.9% NaCl); Gene enrichment analysis using (**F**) NET-GE platform and (**G**) GSEA method showing genes enriched in BCL2L1-gained OvCa cases in TCGA cohort; (**H**) shown was PCR validation of sgRNAs using 3 pairs of primers with Cruser method in SK-OV-3 cells prepared for CRISPR/Cas9 knockout (KO) of BCL2L1; (**I**) western blotting showing BCL-xL level in SK-OV-3 cells with or without BCL2L1-KO; (**J**) cell viability of SK-OV-3 cells with or without BCL2L1-KO plus cisplatin over 5-day course; Western blotting showing (**K**) levels of FGFR/NCAM family members in SK-OV-3 cells with or without BCL2L1-KO and (**L**) BCL2L1 (BCL-xL) level in SK-OV-3 cells with both KD of BCL2L1 and one of FGFR/NCAM family members (N = 5 in all assays; *P < .05; **P < .01; ***P < .001; ****P < .0001).

To further explore the distinction, we first plotted copy number against BCL2L1 expression and found that OvCa with CN gain represented cases with the highest mRNA expression (Figure 2A). We then profiled the drug sensitivity atlas of BCL2L1-gained cancer and found that the genotype was multiresistant to various drugs with no significant sensitivity to any single agent in GDSC (Figure 2B). Notably, BCL2L1-gained cancer was even resistant to BCL-2-targeted agents (Figure 2C). We first showed the expression of BCL2L1 in 3 OvCa cell lines and found that the BCL-xL level was not substantially reduced in BCL2L1-overexpressing SK-OV-3 cells, unlike in OVCAR433 cells (Figure 2D). BCL2L1-overexpressing OVCAR433 cells showed decreased viability following BCL2L1 knockdown (KD), which was synergistically enhanced by cisplatin (Figure 2E). In BCL2L1-independent platinum-resistant A2780 cells, BCL2L1-KD did not alter viability but sensitized cells to cisplatin (Figure 2E). Notably, BCL2L1-transfected SK-OV-3 cells showed unaltered viability following BCL2L1-KD with or without platinum (Figure 2E). By reproducing the TCGA dataset using two methods, we identified activated FGFR/NCAM signaling using the NET-GE method (Figure 2F). GSEA also revealed an overactive FGFR/NCAM pathway (Figure 2G). Thus, we applied CRISPR/Cas9 to knockout (KO) BCL2L1 in SK-OV-3 cells (Figure 2H) and found that the technique could successfully block BCL-xL (Figure 2I). BCL2L1-KO not only significantly decreased cell viability but also synergistically sensitized cells to cisplatin (Figure 2J). We used a KO model to study changes in the FGFR family and found that only FGFR4 levels remained substantially increased by BCL2L1-KO, together with modest NCAM levels (Figure 2K). Next, we used shRNA to block both BCL2L1 and FGFR/NCAM components and found that only targeting FGFR4 substantially decreased the BCL-xL levels (Figure 2L).

### Combined inhibition is required for BCL2L1-gained OvCa

**Figure 3:**
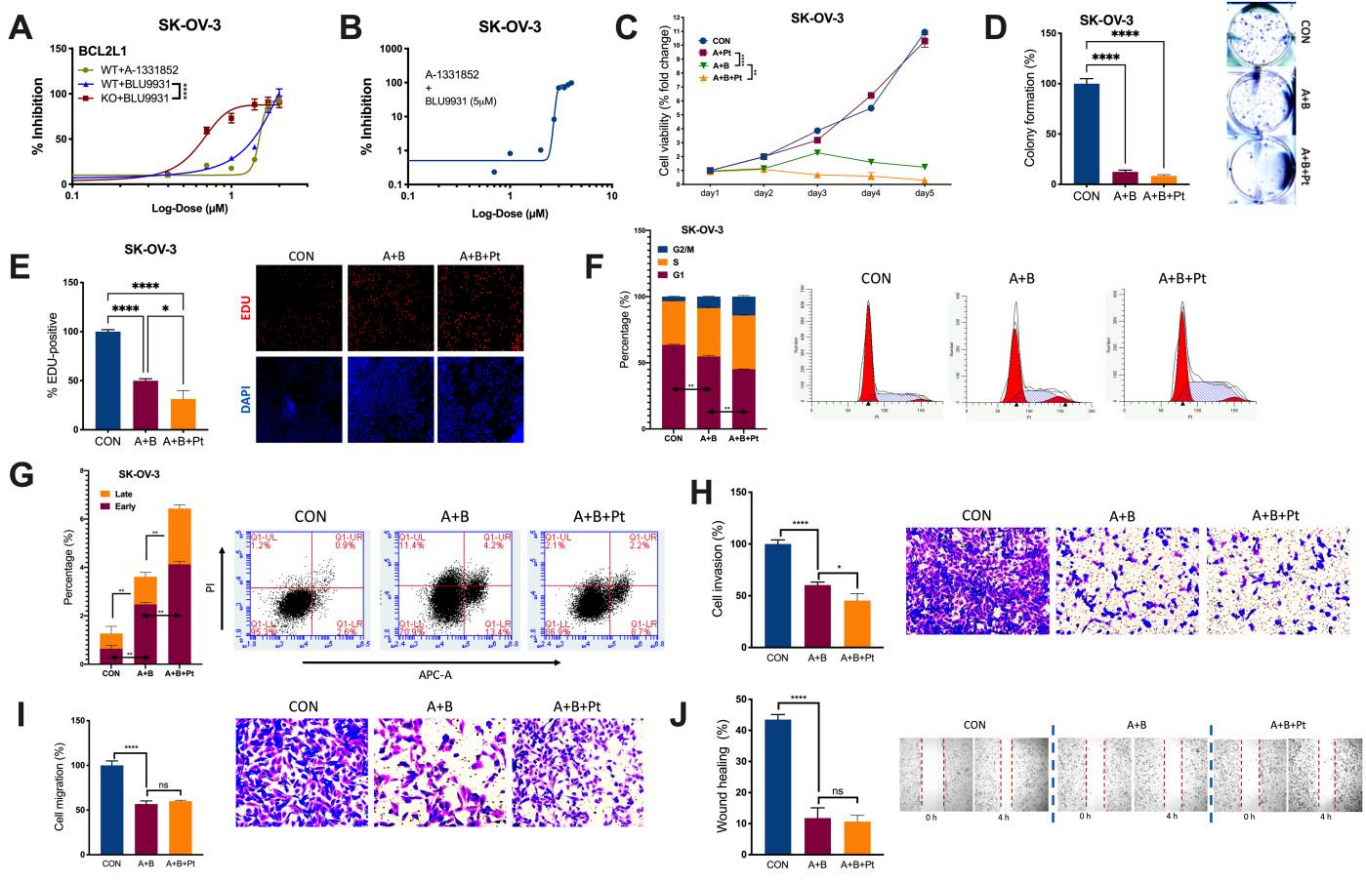
**Combined inhibition is needed for BCL2L1-gained OvCa.** Shown were sigmoidal curve fitting for IC50s of (**A**) A-1331852 and BLU-9931 in SK-OV-3 cells with or without BCL2L1-KO and (**B**) A-1331852 in SK-OV-3 cells treated with set dose of BLU-9931 at 5 μM; (**C**) Cell viability of SK-OV-3 cells treated with A-1331852 (**A**) at 3 μM and/or BLU-9931 (**B**) at 5 μM and/or cisplatin (Pt) at 1 mg/mL in the 5-day course detected by MTT; (**D**) colony formation assay; (**E**) EDU-stained proliferation assay; (**F**) cell cycle profiling and (**G**) apoptosis analysis examined by flow cytometry; Transwell-based (**H**) invasion and (**I**) migration; and (**J**) wound-healing assay in SK-OV-3 cells treated with A-1331852, BLU-9931 and cisplatin all at 72 h of treatment with doses shown in panel (**C**) (N = 5 in all assays; *P < .05; **P < .01; ***P < .001; ****P < .0001).

We examined the IC50s of A and B in OvCa. BCL2L1-KO significantly reduced the IC50 of B from 52.28 μM to 4.33 μM (Figure 3A). Notably, the combination of B at 5 μM significantly reduced the IC50 of A from 31.52 μM to 2.88 μM (Figure 3B). The combination (5 μM of B + 3 μM of A) significantly inhibited the viability of SK-OV-3 cells, and the addition of cisplatin further decreased the viability (Figure 3C). Importantly, the combination suppressed cell fitness in various properties by inhibiting colony formation (Figure 3D), inhibiting EdU-labeled proliferation (Figure 3E), inducing cell cycle arrest (Figure 3F), inducing both early and late apoptosis (Figure 3G), inhibiting cell invasion and migration (Figure 3H, 3I), and impeding wound healing (Figure 3J). Here, we showed that the combination of A and B showed potent inhibition *in vitro*, prompting us to examine its toxicity *in vivo*.

### Dual inhibition of FGFR/BCL-xL is feasible *in vivo*

**Figure 4:**
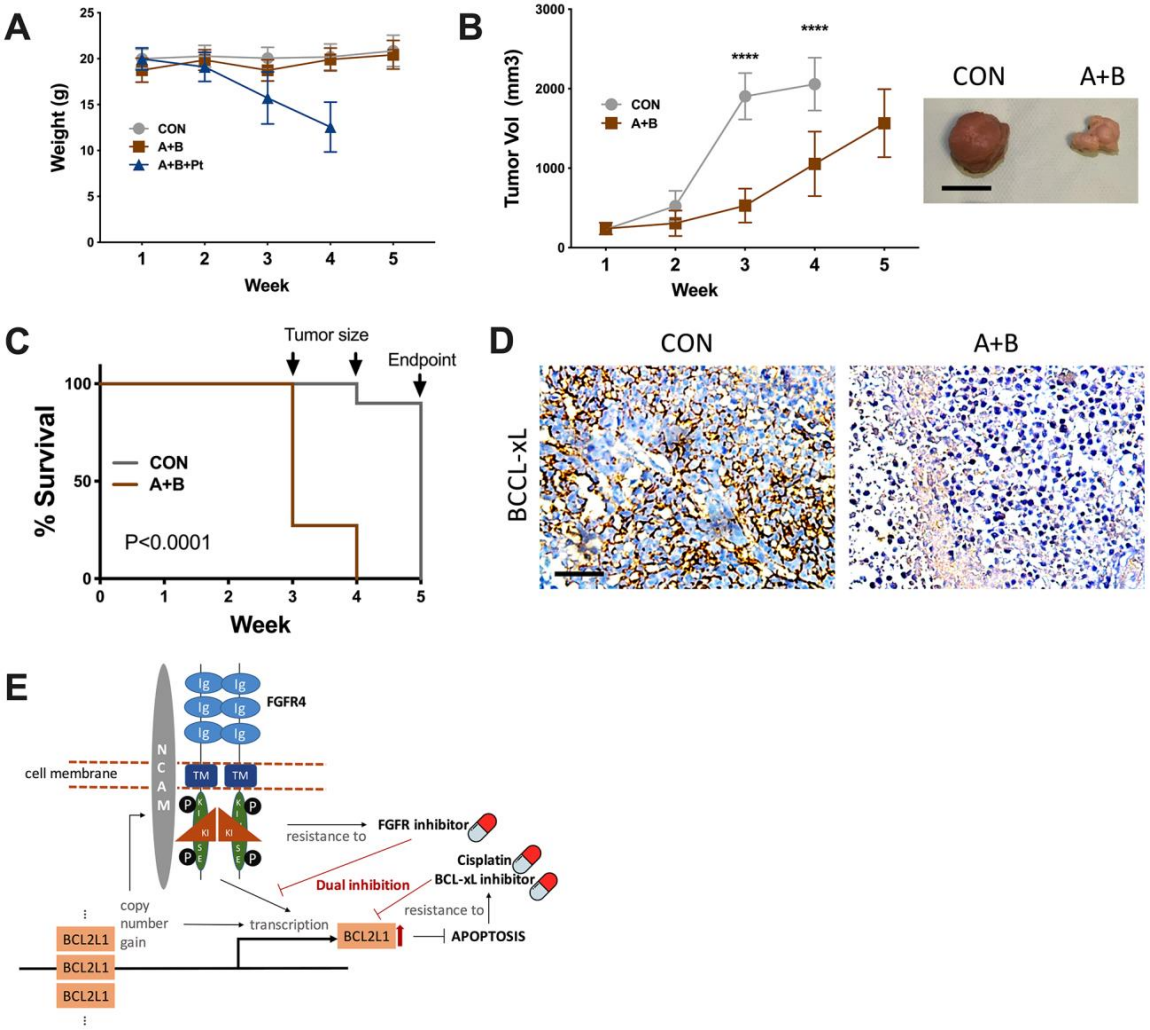
**Dual inhibition of FGFR/BCL-xL is feasible *in vivo*.** (**A**) weight monitoring of SK-OV-3 cell-implanted xenograft mice treated with 25 mg/kg/d of A-1331852, 100-mg/kg of BLU9931, Cisplatin equivalent of 75 mg/m^2^ of dose in humans; (**B**) tumor growth of SK-OV-3 cell-implanted xenograft mice treated with A-1331852 (**A**) and BLU9931 (**B**) or vehicle control with representative tumor image at endpoint, discontinued curve in control group indicating individuals reaching endpoint of tumor size; (**C**) Kaplan-Meier survival curve of control and treatment group compared by Log-rank test; (**D**) representative immunohistochemical staining of BCL-xL in extracted tumors; (**E**) schematic cartoon showing association between FGFR4/NCAM and BCL2L1 with drug sensitivity profile in the setting of current study. (N = 10 in all assays; Scale bar = 1cm; *P < .05; **P < .01; ***P < .001; ****P < .0001).

Although the combination of 3 resulted in a potent anticancer effect *in vitro*, toxicity that caused severe weight loss prevented further testing, whereas dual FGFR/BCL-xL inhibition showed tolerable toxicity compared with the vehicle control (Figure 4A). Over the 5-week course, the control group reached the endpoint of tumor volume at the 4^th^ week, resulting in both significantly larger tumors (Figure 4B) and shortened survival (Figure 4C). IHC of the harvested tumors supported lower BCL-xL levels in the dual inhibition group (Figure 4D).

## DISCUSSION

Enhanced antiapoptotic function that features overexpression and amplification of BCL-2 family members has been implicated in various cancers, including OvCa [15–17]. Unlike in other cancers, BCL-2 family members exert predominantly chemoresistant functions rather than antiapoptotic functions in treatment-naïve patients. This is explicable because truncal genetic and genomic events in OvCa have been sufficiently efficacious in maintain cancer viability, and additional signaling provides no additional selective advantage. Additionally, the interplay and functional redundancy in BCL-2 family proteins result in inconsistent findings regarding a single BCL-2 family protein and its association with a certain phenotype. For example, although BCL-2 protein is associated with lymphatic metastasis and postoperative recurrence [18], the chemoresistant cell line A2780 is not detectable for BCL-2 expression [15]. MCL-1, another BCL-2 family member associated with resistance to BCL-2-targeted agents, also mediates chemoresistance in ovarian cancer [19]. Notably, BCL-xL is the strongest family member that induces chemoresistance, and 40% knockdown of Bcl-xL expression is sufficient to fully activate caspases [20]. These findings also echo our results that BCL2L1 gain and expression showed only marginal prognostic effects. Notably, we found that copy number gain of BCL2L1 demonstrated more resistant overexpression of the gene that can hardly be inhibited by BCL-2-targeted monotherapy, and combined FGFR-targeted therapy is required.

FGFR signaling is also implicated in ovarian cancer [21]. Unlike in other cancers, overactive FGFR signaling is characterized by overexpression rather than mutation in OvCa and interacts with PI3K/AKT signaling to promote cancer progression. Among all members, FGFR4 exerts a major effect in OvCa. Higher FGFR4 expression is associated with a worsened prognosis in OvCa, and targeting FGFR4 significantly decreases the fitness of OvCa *in vitro* and *in vivo*. Additionally, NCAM, the coupling partner to FGFR, plays a critical role in OvCa [22]. Given its important role, several multitarget tyrosine kinase inhibitors, ligand traps and monoantibodies that target FGFR signaling have now entered clinical trials of OvCa [21].

Another interesting finding in the current study is that FGFR/NCAM signaling is activated in BCL2L1-gained OvCa. FGFR4 was previously recognized as an upstream signaling pathway that activates BCL2L1 to combat apoptosis in rhabdomyosarcoma [23]. Dual inhibition of BCL-XL and MCL-1 is required to induce tumor regression in lung squamous cell carcinomas sensitive to FGFR inhibition [14]. Notably, the levels of FGFR4 and NCAM1 were increased in cases of BCL2L1 silencing. Given the multiresistant phenotype of 20q11.21 gain, we postulate that BCL2L1-gained OvCa is inherently characterized by FGFR4 activation and that dual active oncogenic signaling provides cells with intertwined signaling that can resist multiple compounds. In our case, a BCL-xL inhibitor capable of inhibiting the BCL2L1-overexpressing cell line demonstrated a minimal effect on BCL2L1-overexpressing cells, demonstrating substantial BCL-xL levels following treatment. This may result from concomitant FGFR signaling compensating in part for the loss of BCL-xL (Figure 4E).

Platinum is currently the mainstay of medical treatment for OvCa regardless of genotyping. However, chemoresistance is inevitable in most cases and required to overcome with a secondary agent. Reminiscent of this clinical scenario, we found that the dual combination could only combine with cisplatin *in vitro* because of severe toxicity *in vivo*. The addition of a BCL-xL inhibitor has been proposed to sensitize platinum in OvCa [24]. However, according to our findings, only a combination of three inhibitors may work additively, if not synergistically. A better delivery strategy, for instance via nanomedicine [25] to minimize toxicity is urgently needed and warrants further study.
